# Laparoscopic technique and safety experience with barbed suture closure for pelvic cavity after abdominoperineal resection

**DOI:** 10.1186/1477-7819-11-115

**Published:** 2013-05-27

**Authors:** Nobuhisa Matsuhashi, Takao Takahashi, Kenichi Nonaka, Toshiyuki Tanahashi, Hisashi Imai, Yoshiyuki Sasaki, Yoshihiro Tanaka, Naoki Okumura, Kazuya Yamaguchi, Shinji Osada, Kazuhiro Yoshida

**Affiliations:** 1 Surgical Oncology, Gifu University School of Medicine, 1-1 Yanagido, Gifu City 501-1194, Japan

## Abstract

**Background:**

Between April 2005 and December 2012, we performed laparoscopic colorectal resection with regional lymph node dissection on 273 cases of colorectal cancer patients. However, Laparoscopic rectal cancer surgery requires a high degree of skill. Any surgeon who is going to embark on these difficult resections should have at a minimum laparoscopic suturing skills in order to be able to close the peritoneal defect.

**Methods:**

In laparoscopic surgery for rectal cancer, the intracorporeal suture technique required to close the pelvic cavity is very difficult. Barbed sutures have recently been proposed to facilitate laparoscopic suturing. Two patients with rectal cancer who underwent laparoscopic abdominoperineal resection (APR) with intracorporeal closure of the pelvic cavity from September to October 2012 were enrolled in this study.

**Results:**

We present our initial experience of two consecutive cases of intracorporeal closure of the pelvic cavity by totally laparoscopic APR. After clinical follow-up, the two patients have no complaints and have shown no signs of recurrence.

**Conclusions:**

We hypothesized that barbed sutures could potentially improve the efficiency of intracorporeal closure of the pelvic cavity after laparoscopic APR. Further, we expect that use of the V-Loc™ will reduce intra-operative stress on the endoscopic surgeon.

## Background

Laparoscopic colectomy was first reported in 1991. Laparoscopic colectomy has been gradually accepted on the basis of its technical advantages, safety, and feasibility in many studies. However, laparoscopic surgery for rectal cancer is still more complicated than laparoscopic colectomy, owing to its technical difficulty in the pelvis. In laparoscopic surgery for rectal cancer, the intracorporeal suture technique required to close the pelvic cavity is very difficult. The opinion stated in many studies is that it is unnecessary to close the pelvic cavity with laparoscopic abdominoperineal resection (APR). Most colorectal surgeons usually close the pelvic cavity via conversion laparotomy to prevent prolapse of the small intestine into the pelvic cavity.

Barbed sutures have recently been proposed to facilitate laparoscopic suturing. One of these novel sutures, the V-Loc™ 180 (Covidien, Mansfield, MA, USA) consists of a barbed absorbable thread armed with a surgical needle at one end and a loop at the other end, which is used to secure the suture. The barb and loop end make it possible to approximate the tissues without the need to tie surgical knots. To date, the efficacy and suitability of barbed sutures have been reported in gynecologic and urologic surgery. In addition, Lee *et al*. recently reported their use for bowel anastomosis after gastrectomy in humans. We hypothesized that barbed sutures could potentially improve the efficiency of intracorporeal closure of the pelvic cavity after laparoscopic APR. In this article, we present our initial experience of two consecutive cases of intracorporeal closure of the pelvic cavity by totally laparoscopic APR.

## Methods

Two patients with rectal cancer who underwent laparoscopic APR with intracorporeal closure of the pelvic cavity from September to October 2012 were enrolled in this study. Both patients were informed of the study design according to the Ethical Committee on Clinical Investigation of Gifu University Hospital and consented in writing to participate in this study.

In our unit, laparoscopic APR is indicated for rectal cancer up to stage T2N0, whereas in rectal cancers of greater than stage T3 and at N1 advanced stage, open laparotomy is indicated because we add the pelvic side wall lymphadenectomy.

This lymphadenectomy is the traditional procedure in Japan;add in some cases, we adapted APR for rectal cancer with adjusted anal verge, which predicts poor function in elderly patients.

### Patients and clinical evaluations

#### Case 1

**Figure 1:**
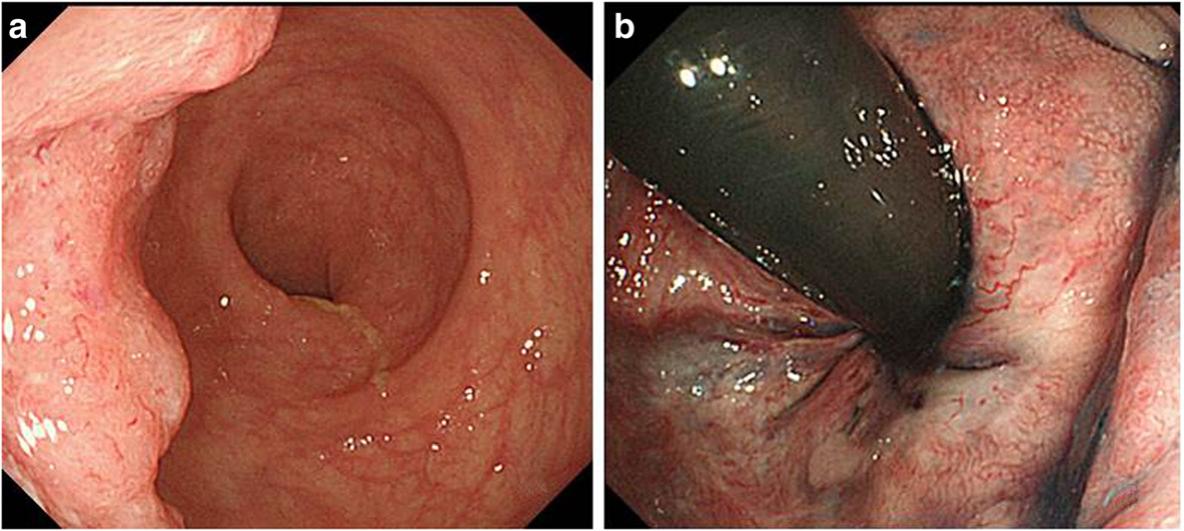
**Case 1: Colonofiberscopy revealed tumor size was 3.5 cm with type 2. a**: straight view, **b **turn-over view.

An 84-year-old man presented with a history of rectal bleeding. He had undergone an orthopedic operation to treat bilateral transformation hip disease. He had taken fondaparinux sodium to prevent blood clots from forming within the blood vessels of his legs. He was diagnosed as having rectal cancer by screening test performed to monitor side effects of this drug. Following biopsy, the lesion was confirmed to be adenocarcinoma. Colonofiberscopy revealed a type 2 tumor of 3.5 cm in size (Figure 1). Staged computed tomography (CT) scans did not reveal distant metastases but did reveal a few swollen lymph nodes in the mesorectum. In consideration of his quality of life in relation to the time necessary to recover anal function, we decided to undertake laparoscopic abdominoperineal resection of the rectum and colostomy formation as best supportive care. Histology revealed stage T3 moderately differentiated carcinoma with extension. The tumor was classified pathologically as a T3N2M0 lesion.

#### Case 2

**Figure 2:**
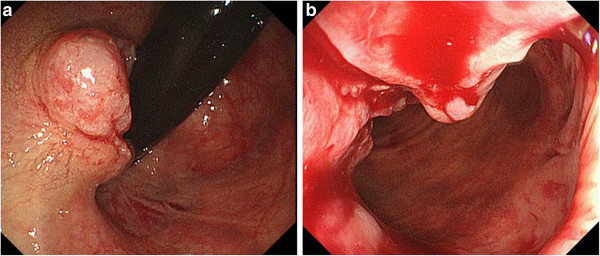
**Case 2: Colonofiberscopy revealed tumor size was 4.5 cm with type 2. **The tumor localized the anal orifice with bleeding. **a**: turn-over view, **b** straight view.

A 59-year-old woman presented with a history of rectal bleeding. She have fallen in primary biliary cirrhosis. Therefore, she have taking with intake steroid drug. Past history revealed hysterectomy for uterine cervical cancer. Colono-fibroscopy revealed a type 2 tumor of 4.5 cm in size (Figure 2). Following biopsy, the lesion was confirmed to be squamous cell carcinoma. Staged CT scans did not reveal any lymph node swelling or distant metastases. Radiotherapy was separated from adaptation because she continued oral intake of the steroid drug.

The patient successfully underwent laparoscopic APR of the rectum and colostomy formation. Histology revealed squamous cell carcinoma.

#### V-Loc™ barbed sutures

**Figure 3:**
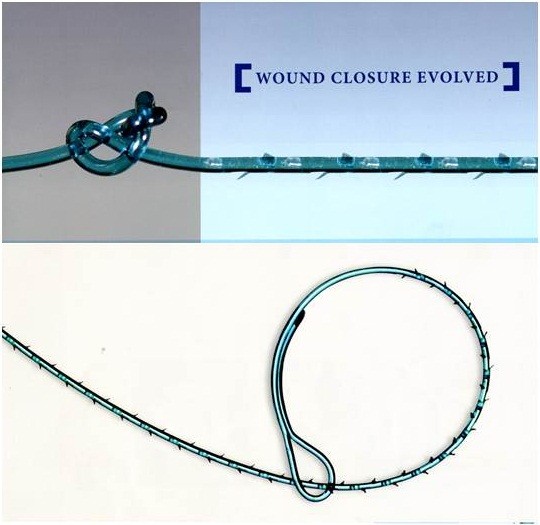
**A total of 30 cm of 3–0 V-Loc 180 suture on a V-20 needle (26 mm tapered).** No barbs in the first 2 cm of the suture, allowing for readjustment of the throw without adverse effects, and a loop at the other end for passing the needle to secure the suture.

The V-Loc™ 180 closure device is a unidirectional barbed variant of the absorbable copolymer polyglyconate (Maxon; Covidien Mansfield, MA, USA) and has the same material and degradation properties as Maxon (monofilament polyglyconate) suture. Tissue closing strength is approximately 50% at 30 days, with complete absorption in 180 days (Figure 3). Although etching of the barbs reduces the core diameter of these sutures, they have been sized according to their post-etching diameter, and 3–0 V-Loc™ suture has the same tensile strength as 3–0 Maxon. A loop at the end of the suture can be used for knotless suturing, and the first 2 cm of the suture are without barbs to allow throws to be readjusted before the barbs are engaged.

#### Patients and operative procedure

The operation was performed according to the following seven procedural steps. First, laparoscopic exploration was performed with five ports after pneumoperitoneum was induced. If necessary, intraoperative excisional biopsy or cytological examinations were performed. Second, mobilization of both the jejunum and the ileum was done in right-head-ventral side. This mobilization was indicated to provide a good operative view of the left side of the colon. Our devise performed to put on each half gauze near the ligament of Treitz and right iliac artery.This reason is not so good with operation’s view in order to the intervention of small intestine Dissection of the left side of the colon was performed from a medial-to-lateral retroperitoneal approach.

Third, lymphadenectomy around the inferior mesenteric artery and ligation of this artery were performed. Retroperitoneal dissection was performed from a medial-to-lateral approach. Fourth, mobilization of the rectum and excision of the mesorectum were performed. We accessed the deepest part of the pelvic cavity by retracting the rectum toward the dorsal side. We identify the levator ani muscle as a device at this time and excise a portion of this muscle from the abdominal cavity. We consider it very useful to continue between the vulval orifice and intra-abdominal cavity. At this point in the procedure, the proximal rectum (recto-sigmoid section, Rs) is cut with a stapling device (Endo GIA™ with Tri-Staple™ Technology; Covidien Mansfield, MA, USA).

Fifth, with the help of the perineal surgeon, the rectum together with the whole mesorectum was fully mobilized, and the specimen was retrieved through the perineal wound. The perineal wound was closed primarily in the pelvic cavity via a separate stab wound. Sixth, we performed the new technique of total laparoscopic APR with intracorporeal closure of the pelvic cavity. Using a 30-cm 3–0 V-Loc™ 180 suture on a V-20 needle (26-mm tapered) we start closure of the full-thickness peritoneal tissue layer from the corner of the pubis to toward the sacrum with a continuous technique. After the last stitch, the suture is simply cut, without the need for any knots to anchor the last throw. Intracorporeal closure of the pelvic cavity is then accomplished. Seventh, an end colostomy was fashioned at the left lower quadrant port site after a 2-cm disk of skin was excised. Laparoscopic APR is a perfect finish to the total laparoscopic intracorporeal procedure.

## Results

**Figure 4:**
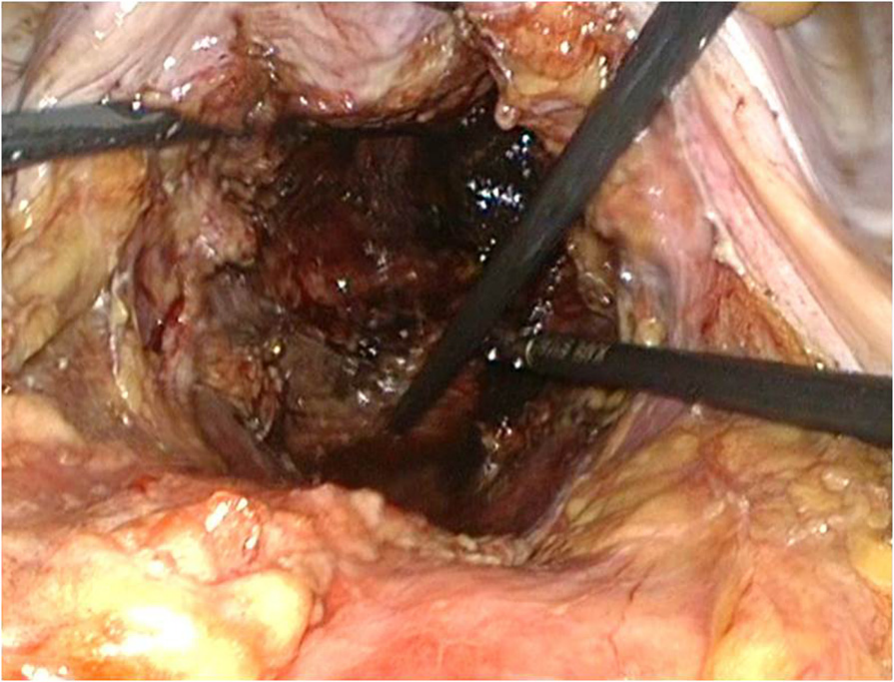
Case 1: Intra-abdominal view of bowel loops mobilized from perineal wall defect.

**Figure 5:**
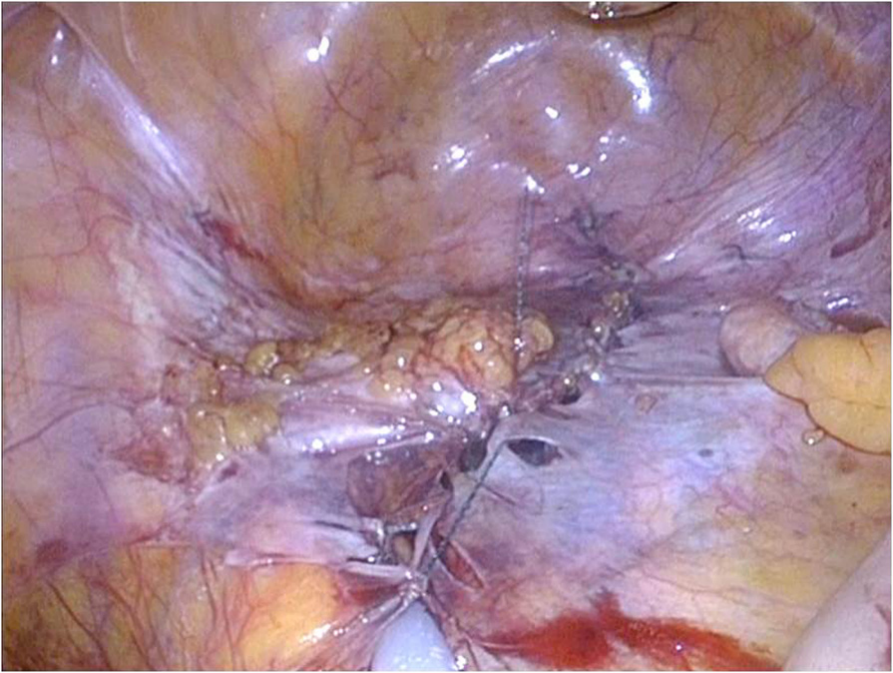
Case 1: Intra-abdominal view showed with intracorporeal closing of pelvic cavity by the V-Lock.

**Figure 6:**
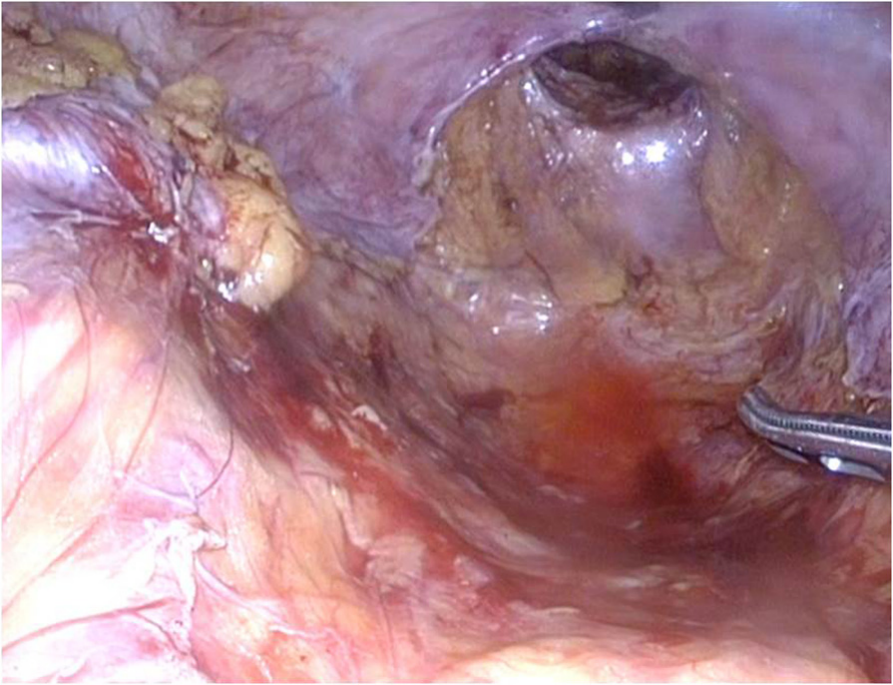
Case 2: Intra-abdominal view of bowel loops mobilized from perineal wall defect.

**Figure 7:**
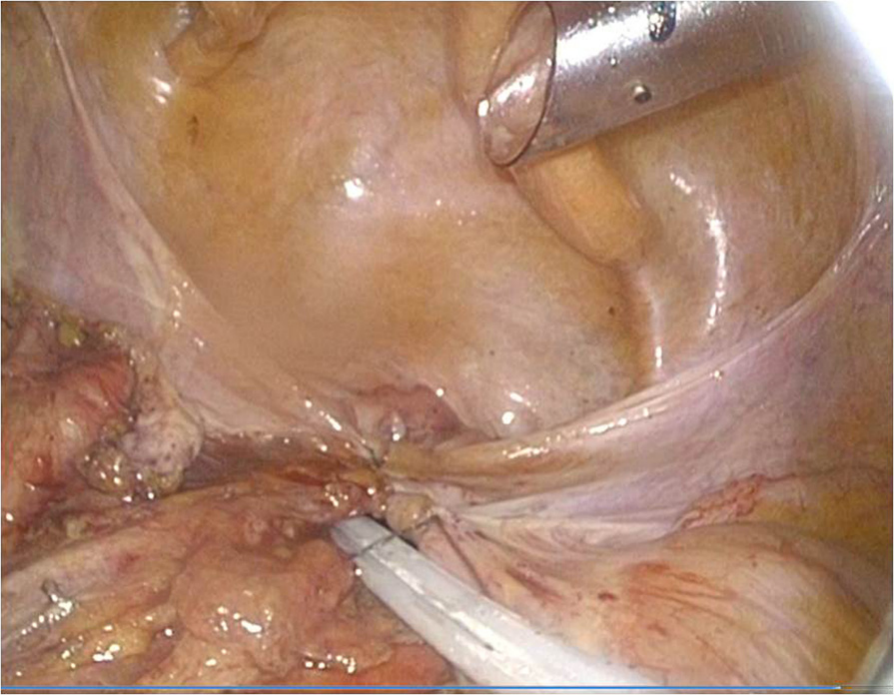
Case 2: Intra-abdominal view showed with intracorporeal closing of pelvic cavity by the V-Lock.

Patient demographics and postoperative outcomes are shown in Table 1. In the present study, one man and one woman underwent laparoscopic APR with intracorporeal closing of the pelvic cavity using the knotless, unidirectional, barbed suture device. The procedure in both patients was successfully performed with no intra-operative complications, and no conversion to other procedures was required. Mean total operation time was 301 and 330 minutes and mean intracorporeal closing suture time was 21.5 minutes (range 18 to 25 minutes). Both patients tolerated a liquid diet on the day following the operation (Case 1: Figures 4, 5; Case 2: Figure 6, 7).

**Table 1:** Patient demographics and operative outcomes

**Characteristics**	**Case 1**	**Case 2**
Sex	M	F
Age	84	58
Body mass index, kg/m^2^	23.6	23.4
Closing time, min	18	25
Operating time, min	301	330
Blood loss, ml	120	70

## Discussion

Between April 2005 and December 2012, we performed laparoscopic colorectal resection with regional lymph node dissection on 273 cases colorectal cancer patients. In general, APR is associated with higher rates of cancer recurrence compared with anterior resection [1]. This is because more advanced and lower rectal cancers are treated with this operation [2-5]. Although the introduction of total mesorectal excision has helped to reduce the rate of cancer recurrence after anterior resection, the apparent obstacle for APR remains almost unchanged, amounting to a recurrence rate of more than 15% [6-10]. The perioperative complications that were assessed included inadvertent intra-abdominal injury and accidental bleeding. The commonly observed cardiorespiratory and wound-related complications, and the need for reoperation, were analyzed as postoperative complications [11]. Re-operation was mostly required for postoperative intestinal obstruction.

But the frequent postoperative complication will be intestinal obstruction caused by the protrusion of the pelvic cavity. Many colorectal surgeons admit that mesenteric or peritoneal reconstruction is not always performed with laparoscopic rectal surgery. But no peritoneal reconstruction will result in an increase in the occurrence of the intestinal obstruction; thus, synchronous peritoneal repair is recommended for patients undergoing laparoscopic APR [12]. However, in the pelvic cavity, suturing and knot tying are difficult because of its depth and the inconvenience of accessing the pelvic cavity. We consider that suturing and knot tying may be easy for experienced laparoscopic surgeons, but we are afraid that only experienced laparoscopic surgeons with a high-level technique will perform as recommended with suturing and knot tying; we think that suturing and knot tying with high level technique in the pelvic cavity is not generally widespread in hospitals.

However, our experience in peritoneal repair with the V-Loc™ suture indicated that suturing technique is only required for this procedure and a special technique is not required with the V-Loc™ [13-16].

In addition, we believe that the medical treatment costs can be reduced when this continuous suture method requiring only one suture is used. A very strong advantage of using the V-Loc™ is in the prevention of postoperative complications [17-20].

Although Lee *et al*. first reported the incorporation of a knotless, unidirectional, barbed suture into the staple-conserving technique for delta-shaped gastroduodenostomy after laparoscopic distal gastrectomy in the domain of digestive surgery [21-23], there are no reports of its use in the domain of colorectal surgery. In our initial experience, we applied a knotless, unidirectional, barbed suture to the intracorporeal closure of the pelvic cavity with laparoscopic APR. This suture enabled the surgeon to work efficiently with both hands and to focus exclusively on subsequent stitch placement, without the need to maintain tension on the preceding throws to prevent slippage. In addition, in this small patient series, mean suturing time was 21.5 minutes. The V-Loc™ 180 absorbable wound closure device was initially studied for skin closure. Subsequent studies on porcine enteric anastomoses by Demyttenaere and colleagues [23] demonstrated not only closure equivalence with Maxon and equivalent histopathologic inflammatory response at 3, 7, and 14 days but faster anastomosis times. To date, this barbed suture device has had limited application in gynecologic and urologic surgery. Our study is the first, to our knowledge, to report the use of the knotless, unidirectional, barbed suture for intracorporeal closure of the pelvic cavity in laparoscopic colorectal surgery, and we noted several advantages of this device for intracorporeal pelvic cavity closure.

## Conclusions

In conclusion, although the impact of this new closure device on laparoscopic APR still needs to be evaluated in detail, use of the knotless, unidirectional, barbed suture appears to be safe, feasible, and efficacious for intracorporeal closure of the pelvic cavity after laparoscopic APR. Further, we expect that use of the V-Loc™ will reduce intra-operative stress on the endoscopic surgeon.

### Consent

Written informed consent was obtained from the patient for publication of this case report and accompanying images.

## Competing interests

The authors declare that they have no competing interests.

## Authors’ contributions

Study conception and design: NM, TT. Acquisition of data: NM, TT, KN, HI, YS, YT, NO, KY, and SO. Analysis and interpretation of data: NM. Drafting of manuscript: NM. Critical revision: NM, TT, and KY. Supervision: KY. All authors read and approved the final manuscript.
